# The Interaction of lncRNA XLOC-2222497, *AKR1C1,* and Progesterone in Porcine Endometrium and Pregnancy

**DOI:** 10.3390/ijms21093232

**Published:** 2020-05-02

**Authors:** Tao Su, Haile Yu, Gan Luo, Mengxia Wang, Changfan Zhou, Long Zhang, Bin Hou, Chi Zhang, Min Liu, Dequan Xu

**Affiliations:** 1 Key Laboratory of Swine Genetics and Breeding of Ministry of Agriculture and Rural Affairs, Huazhong Agricultural University, Wuhan 430070, China; 15607126210@163.com (T.S.); master_yuhaile@163.com (H.Y.); logan19941115@163.com (G.L.); xingxing139139139@163.com (M.W.); 13341404358@163.com (C.Z.); 18763821961@163.com (L.Z.); hzau_houbin@sina.com (B.H.); ljames2324@163.com (C.Z.); 2 Key Laboratory of Agricultural Animal Genetics, Breeding and Reproduction of Ministry of Education, Huazhong Agricultural University, Wuhan 430070, China; 3 Colleges of Animal Science & Technology, Huazhong Agricultural University, Wuhan 430070, China; 4 College of Veterinary Medicine, Huazhong Agricultural University, Wuhan 430070, China

**Keywords:** *AKR1C1*, endometrium, pig, lncRNA, progesterone

## Abstract

The endometrium is an important tissue for pregnancy and plays an important role in reproduction. In this study, high-throughput transcriptome sequencing was performed in endometrium samples of Meishan and Yorkshire pigs on days 18 and 32 of pregnancy. Aldo-keto reductase family 1 member C1 (*AKR1C1*) was found to be a differentially expressed gene, and was identified by quantitative real-time PCR (qRT-PCR) and Western blot. Immunohistochemistry results revealed the cellular localization of the AKR1C1 protein in the endometrium. Luciferase activity assay demonstrated that the *AKR1C1* core promoter region was located in the region from −706 to −564, containing two nuclear factor erythroid 2-related factor 2 (NRF2) binding sites (antioxidant response elements, AREs). *XLOC-2222497* was identified as a nuclear long non-coding RNA (lncRNA) highly expressed in the endometrium. *XLOC-2222497* overexpression and knockdown have an effect on the expression of *AKR1C1*. Endocrinologic measurement showed the difference in progesterone levels between Meishan and Yorkshire pigs. Progesterone treatment upregulated *AKR1C1* and *XLOC-2222497* expression in porcine endometrial epithelial cells. In conclusion, transcriptome analysis revealed differentially expressed transcripts during the early pregnancy process. Further experiments demonstrated the interaction of XLOC-2222497/AKR1C1/progesterone in the endometrium and provided new potential targets for pregnancy maintenance and its control.

## 1. Introduction

Pregnancy is a key physiological process affecting reproduction [1]. Successful pregnancy requires intricate bidirectional regulation between embryo and the matrix [2]. The pig (*Sus scrofa*) is one of the most important domesticated animals and is also well-suited as a biomedical model because of similarities in anatomy and physiology between pigs and humans [3,4]. Days 18 and 32 of pig pregnancy are the two peaks of embryonic loss [5,6]. Although the two breeds have a similar number of ovulations, Meishan pigs (MS) have a higher litter size than Yorkshire pigs (YK) due to the high embryo survival rate of Meishan pigs [7,8]. Therefore, it is essential to explore the regulation mechanism of endometrium changes and the genetic difference between the two breeds during the two periods.

Long non-coding RNAs (lncRNAs) are non-protein-coding RNA species longer than 200 nucleotides [9,10]. Many lncRNAs were reported to regulate many biological processes via regulating the expression of target genes at transcriptional and post-transcriptional levels [11]. Some lncRNAs can function as signals, decoys, guides, and scaffolding in regulating nuclear activities [12]. Some lncRNAs have participated in the regulation of the cell cycle [13,14], cell differentiation [15,16], and cell apoptosis [17,18,19]. Some lncRNAs have key roles in cancer progression [20,21], and can also serve as diagnostic or prognostic markers [22,23].

Recent studies have also revealed that several lncRNAs play important roles in reproduction. The studies found that the meiotic recombination hot spot locus (Mrhl, a type of single-axon lncRNA) regulated spermatogenesis through two molecular mechanisms [24]. Yerushalmi et al. found 89 lncRNAs, 12 of which are encoded within introns of genes involved in granulosa cell processes [25]. Nakagawa et al. found that *NEAT1*-knocked-out mice with normal ovulation were stochastically infertile and *NEAT1* is essential for corpus luteum formation and the pregnancy under suboptimal conditions [26]. Rosalia et al. found that 41 lncRNAs could interact with oocyte microRNAs (miRNAs) and may regulate folliculogenesis [27]. lncRNA-*TCL6* promotes early abortion and inhibits placenta implantation via the epidermal growth factor receptor (EGFR) pathway [28]. Downregulation of lncRNA-*H19* could inhibit ectopic endometrial cell proliferation and invasion by modulating *miR-124-3p* and *ITGB3*, offering a novel target for treatment of endometriosis [29]. lncRNA *AK124742* may be a biomarker to predict pregnancy [30]. These findings are important in both basic reproductive research and clinical application.

Several hormones such as estrogen [31], progesterone [32,33], prostaglandin E, and prostaglandin F [34,35] are involved in the regulation of the pregnancy process. Aldo-keto reductases (AKRs) are part of the oxidoreductase super family and play an important role in the cellular response to electrophilic, osmotic, and oxidative stress, depending on the presence of the coenzyme nicotinamide adenine dinucleotide phosphate (NADPH) [36,37]. The proteins encoded by *Akr* genes catalyze a variety of metabolic oxidation–reduction reactions, ranging from the reduction of glucose, glucocorticoids, and small carbonyl metabolites to glutathione conjugates and phospholipid aldehydes. Substrates of the family include glucose, steroids, glycosylation end products, lipid peroxidation products, and environmental pollutants [38]. The aldo-keto reductase type 1C (AKR1C), part of the AKR superfamily, comprises the isoforms AKR1C1-AKR1C4 that catalyze NADPH-dependent reductions and have been implicated in biosynthesis, intermediary metabolism, and detoxification [39]. They serve important roles in the metabolism of steroid hormones, conjugated steroids, neurosteroids, and bile acids [40,41]. AKR1C genotypes were associated with nipple number as well as possible effects on age at puberty and ovulation rate in pigs [42]. Aldo-keto reductase family 1 member C1 (*AKR1C1*), which possesses 20α-Hydroxysteroid dehydrogenase (20α-HSD) activity, is associated with numerous important biological processes [38,43], and has crucial roles in the biosynthesis and inactivation of all classes of steroid hormones, and also in the biosynthesis of neurosteroids and prostaglandins [44]. Knockout of the gene encoding AKR1C1 in the mice resulted in decreasing the number of pups and prolonging the durations of the estrous cycle, pseudopregnancy, and pregnancy [45].

In this study, high-throughput transcriptome sequencing was performed in endometrium samples of Meishan and Yorkshire pigs on days 18 and 32 of pregnancy. Differentially expressed transcripts (including mRNAs and lncRNAs) were identified. Further experiments demonstrated that *XLOC-2222497* regulated *AKR1C1* in porcine endometrial epithelial cells, and may play an important role in the pregnancy process.

## 2. Results

### 2.1. Analysis of lncRNAs and mRNAs in the Endometrium

**Figure 1:**
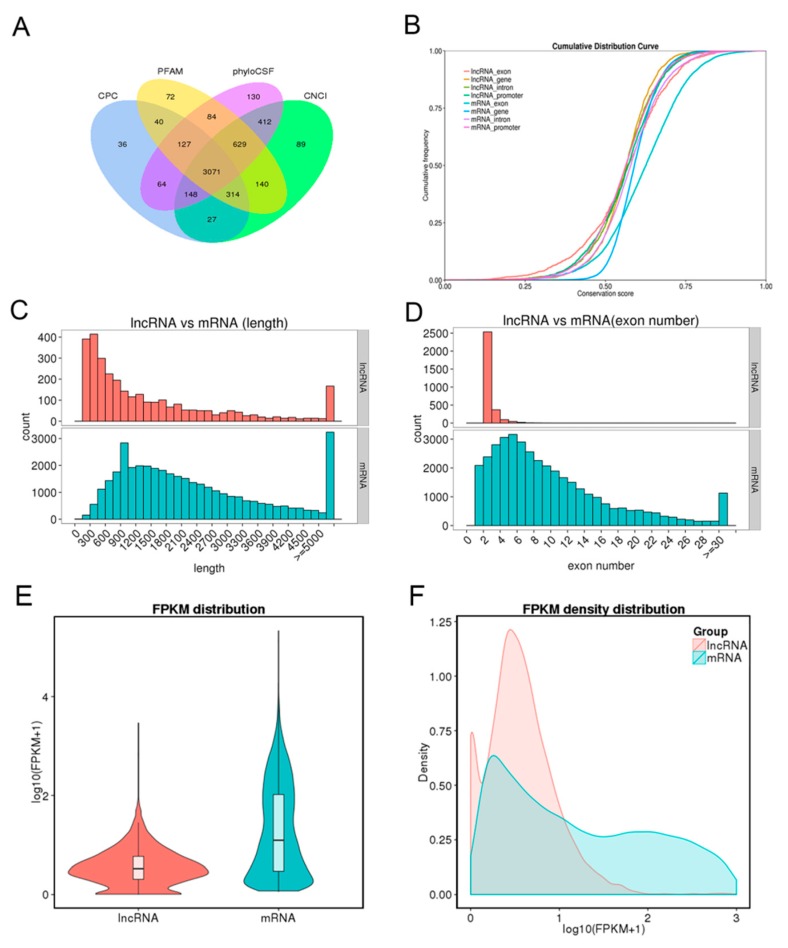
Long non-coding RNA (lncRNA) identification and comparative analysis. (**A**) Identification of lncRNAs by coding potential calculator (CPC), protein families (Pfam), phylogenetic codon substitution frequency (PhyloCSF), and coding-non-coding index (CNCI) softwares. (**B**) Conservation analysis of lncRNAs and mRNAs. (**C**) Distribution of transcript length. Red for lncRNAs and cyan for mRNAs. (**D**) Distribution of exon number per transcript. Otherwise, as in C. (**E**) Fragments per kilobase per million (FPKM) distribution of lncRNAs and mRNAs. (**F**) FPKM density distribution of lncRNAs and mRNAs.

To identify the long non-coding RNAs (lncRNAs) and messenger RNAs (mRNAs), eight cDNA libraries were constructed for RNA-seq from endometrium samples of Meishan and Yorkshire pigs on days 18 and 32 of pregnancy. Coding potential calculator (CPC) analysis, coding-non-coding index (CNCI) analysis, protein families (Pfam) protein domain analysis, and phylogenetic codon substitution frequency (PhyloCSF) analysis were performed to identify lncRNAs. As shown in Figure 1A, 3071 lncRNAs were identified in the intersection of the data from four analysis methods. Expression levels of all transcripts were calculated by the HTseq software, and the expression abundance was described by the reads per kilobase per million reads (RPKM). Several highly expressed lncRNAs with RPKM >100 were found in the eight libraries; most of these identified lncRNAs were shared among different libraries (Supplementary Table S1).

Conservation analysis between lncRNAs and mRNAs showed the conservation of mRNAs, especially in exon regions, was higher than that of lncRNAs (Figure 1B). Comparison analysis of structure indicated that the transcript length and exon number of lncRNA were less than those of mRNAs (Figure 1C,D). Fragments per kilobase per million reads (FPKM) were calculated to perform expression compare analysis between lncRNAs and mRNAs. As shown in Figure 1E,F, mRNAs had higher expression levels than lncRNAs. At the same time, 1535 differentially expressed mRNAs and 278 differentially expressed lncRNAs were identified in the comparison of Yorkshire pigs on days 32 of pregnancy (YK32) vs. Yorkshire pigs on days 18 of pregnancy (YK18) (Supplementary Table S2 and Table S3).

### 2.2. Feature Identification of AKR1C1

**Figure 2:**
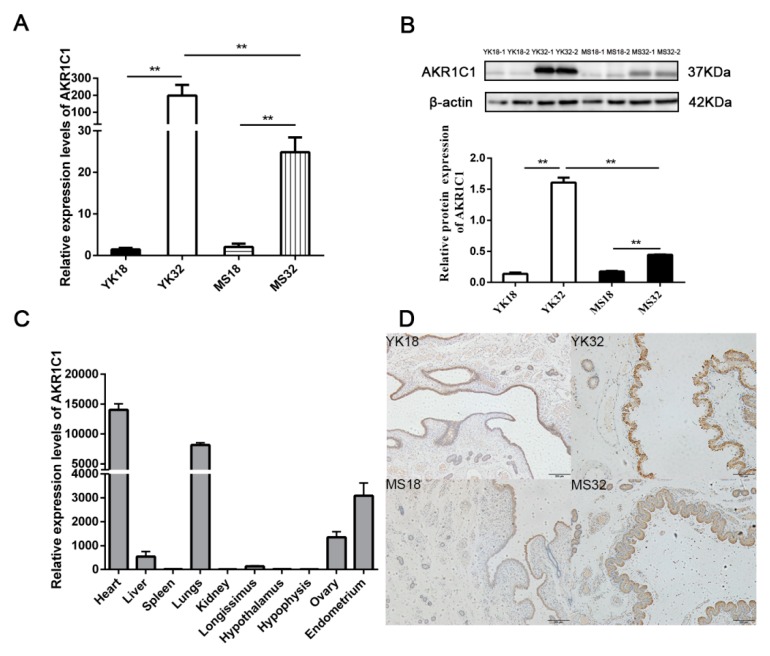
Feature identification of *AKR1C1*. (**A**) RNA expression profile of *AKR1C1* in the endometrium from Meishan and Yorkshire pigs on days 18 and 32 of pregnancy by qRT-PCR. ** represents *p* < 0.01. (**B**) Protein expression profile of *AKR1C1* in the endometrium from Meishan and Yorkshire pigs on days 18 and 32 of pregnancy by Western blot. Down diagram: quantification of Western blot results, ** represents *p* < 0.01. (**C**) Expression profile of *AKR1C1* by qRT-PCR in different tissues. (**D**) Immunohistochemistry result of AKR1C1 in the endometrium.

*AKR1C1* was found to be a differentially expressed gene in the comparisons of YK32 vs. YK18 and Meishan pigs on days 32 of pregnancy (MS32) vs. Meishan pigs on days 18 of pregnancy (MS18). qRT-PCR and Western blot were used to confirm the expression profile of *AKR1C1* (Figure 2A,B). The results revealed that *AKR1C1* had high expression levels in 32–day pregnant endometrium, especially in YK32 samples. The tissue expression profile of *AKR1C1* was also analyzed by qRT-PCR. As shown in Figure 2C, *AKR1C1* had high expression levels in heart, lungs, endometrium, and ovary. Simultaneously, immunohistochemistry results revealed that AKR1C1 protein was located in the endometrium epithelium, including lumen epithelium and glandular epithelium (Figure 2D).

### 2.3. Identification of AKR1C1 Gene Promoter Region

**Figure 3:**
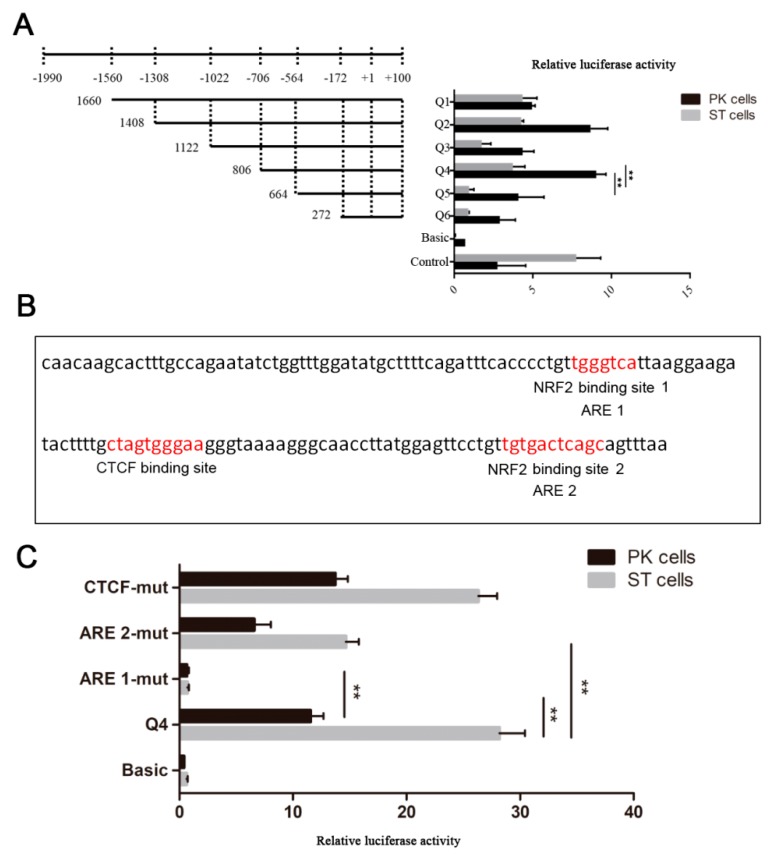
Identification of *AKR1C1* gene promoter region. (**A**) Luciferase activity assay of *AKR1C1* gene promoter. Left: schematic diagram of recombinant plasmids. Right: luciferase activity assay of recombinant plasmids in pig kidney cells (PK cells) and swine testis cells (ST cells). pGL3-basic plasmid was used as negative control. pGL3-control plasmid was used as positive control. ** represents *p* < 0.01. (**B**) Transcription factor prediction of *AKR1C1* core promoter region. Red means potential binding sites of transcription factor. (**C**) Luciferase activity assay of site mutant plasmid. pGL3-basic plasmid was used as negative control. Q4 plasmid was used as positive control. ** represents *p* < 0.01.

To identify the possible promoter region of *AKR1C1* gene, the 1660bp 5’ flanking sequence fragment of *AKR1C1* gene was amplified. Then, a series of deletion reporter plasmids (Q1–Q6) were constructed (Figure 3A) and transfected into pig kidney (PK) cells and swine testis cells (ST cells) for 24–48 h to analyze the promoter activity (where + 1 was the transcription start site). As shown in Figure 3A, the luciferase activities of Q5 (−564~ + 100) and basic were extremely significantly lower than that of Q4 in both PK and ST cells (−706~ + 100) (*p* < 0.01). This result implied that the *AKR1C1* core promoter region was in the fragment from −706 to −564. The luciferase activity of Q3 (−1022~ + 100) was extremely significantly lower than that of Q4 (−706~+100) (*p* < 0.01), which implied that there were negative regulatory elements (NRE) in the region from −1022 to −706.

The transcription factor binding to the *AKR1C1* core promoter region was predicted by JASPAR (http://jaspar.genereg.net/). There were one CCCTC-binding factor (CTCF) binding site and two antioxidant response elements (AREs) in this region (Figure 3B). Nuclear factor erythroid 2-related factor 2 (NRF2, which is also known as NFE2) was predicted to bind to the two AREs. In this study, there were two NRF2 binding sites in the *AKR1C1* core promoter region. When ARE 1 and 2 were mutated (ARE 1-mut, ARE 2-mut), the luciferase activity decreased extremely significantly in ST cells (*p* < 0.01)(Figure 3C). In PK cells, the luciferase activity of ARE 1-mut was also extremely significantly lower than that of Q4 (*p* < 0.01). These results demonstrated NRF2 may play an important role in the transcriptional regulation of *AKR1C1*.

### 2.4. Feature Identification of XLOC-2222497

**Figure 4:**
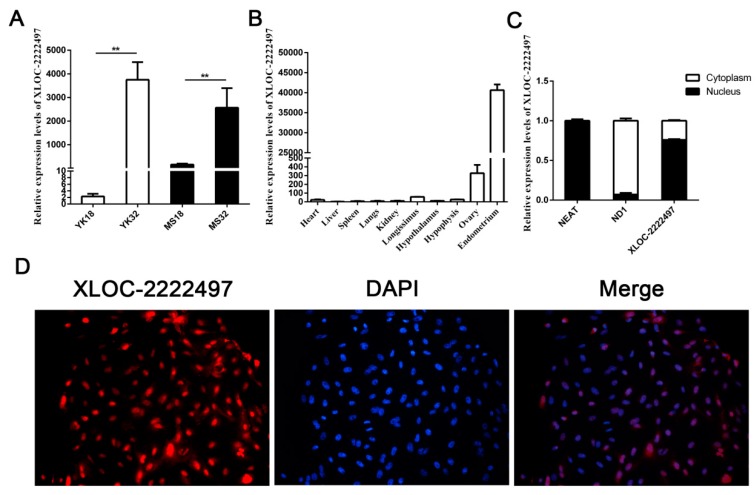
Feature identification of *XLOC-2222497*. (**A**) RNA expression profile of *XLOC-2222497* in the endometrium from Meishan and Yorkshire pigs on days 18 and 32 of pregnancy by qRT-PCR. ** represents *p* < 0.01. (**B**) Expression profile of *XLOC-2222497* in different tissues. (**C**) qRT-PCR results of *XLOC-2222497* cell-fractionation assay. (**D**) RNA fluorescence in situ hybridization (FISH) results of *XLOC-2222497* for subcellular localization. Red fluorescent probe: *XLOC-2222497*; blue fluorescent probe: 4’, 6-diamidino-2-phenylindole (DAPI).

*XLOC-2222497* was a differentially expressed lncRNA in our RNA-seq data. qRT-PCR results confirmed its expression profile (Figure 4A). *XLOC-2222497* had high expression levels in 32-day pregnant endometrium. The expression level of *XLOC-2222497* in Meishan pigs was higher than that in Yorkshire on day 18 of pregnancy. The tissue expression profile revealed that *XLOC-2222497* was highly expressed in the endometrium (Figure 4B). To investigate the subcellular localization of *XLOC-2222497*, the fluorescent probes of *XLOC-2219602* were transfected into porcine endometrial epithelial cells for RNA fluorescence in situ hybridization (FISH). As shown in Figure 4D, *XLOC-2222497* was mainly located in the nucleus. Cell-fractionation assay obtained the same result (Figure 4C). To predict the coding potential of XLOC-2222497, online software CPC (http://cpc.cbi.pku.edu.cn) was used. As shown in Table 1, XLOC-2222497 had no coding potential.

### 2.5. The Regulation of XLOC-2222497 on AKR1C1

**Figure 5:**
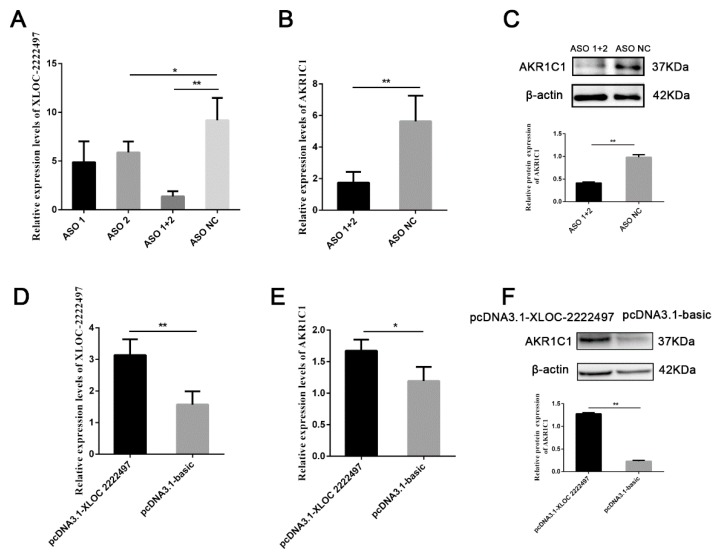
The regulation of *XLOC-2222497* on *AKR1C1*. (**A**) qRT-PCR results of antisense oligonucleotide (ASO) knockdown efficiency detection. * represents *p* < 0.05. ** represents *p* < 0.01. (**B**) qRT-PCR results of *AKR1C1* after the transfection of ASOs. ** represents *p* < 0.01. (**C**) Western blot results of AKR1C1 after the transfection of ASOs. Down diagram: quantification of Western blot results, ** represents *p* < 0.01. (**D**) qRT-PCR results of *AKR1C1* overexpression efficiency detection. ** represents *p* < 0.01. (**E**) qRT-PCR results of *AKR1C1* after the transfection of pcDNA3.1–XLOC-2222497. * represents *p* < 0.05. (**F**) Western blot results of AKR1C1 after the transfection of pcDNA3.1–XLOC-2222497. Down diagram: quantification of Western blot results, ** represents *p* < 0.01.

To identify the function of *XLOC-2222497*, antisense oligonucleotide (ASO) was used to decline the expression of *XLOC-2222497*. As shown in Figure 5A, *XLOC-2222497* expression levels were extremely significantly inhibited after the transfection of ASO1 + 2. ASO1 + 2 combined treatment was used in a further experiment and inhibited AKR1C1 mRNA (Figure 5B) and protein (Figure 5C) expression. To further investigate the effect of *XLOC-2222497* on *AKR1C1*, an overexpression plasmid of *XLOC-2222497* was constructed (pcDNA3.1–XLOC-2222497) and transfected into porcine endometrial epithelial cells. As shown in Figure 5D, the overexpression of *XLOC-2222497* significantly promoted the expression of *AKR1C1* at the mRNA level (Figure 5E) and protein level (Figure 5F). These results demonstrated the positive regulation of *XLOC-2222497* on *AKR1C1*.

### 2.6. Progesterone Measurement and Regulation on XLOC-2222497 and AKR1C1

**Figure 6:**
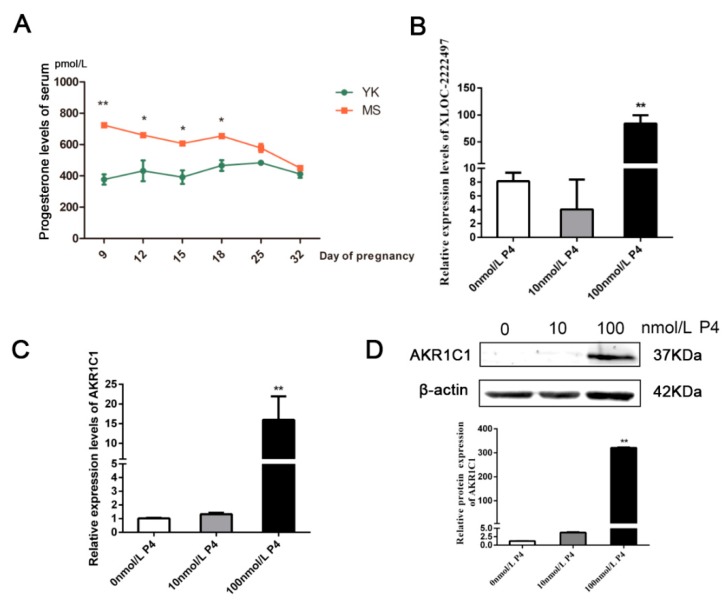
Progesterone measurement and regulation on *XLOC-2222497* and *AKR1C1*. (**A**) Progesterone levels in serum of Meishan and Yorkshire pigs at different pregnancy stages. * represents *p* < 0.05, ** represents *p* < 0.01,. (**B**) Expression levels of *XLOC-2222497* after the treatment of progesterone in porcine endometrial epithelium cells. ** represents *p* < 0.01. (**C**) RNA expression levels of *AKR1C1* after the treatment of progesterone in porcine endometrial epithelium cells. ** represents *p* < 0.01. (**D**) Protein expression levels of AKR1C1 after the treatment of progesterone in porcine endometrial epithelium cells. Down diagram: quantification of Western blot results, ** represents *p* < 0.01.

*AKR1C1* plays a key role in the progesterone metabolism process [40]. Therefore, progesterone levels were measured in the serum of Meishan and Yorkshire pigs in different early pregnancy stages (Figure 6A). On days 9, 12, 15, 18 of pregnancy, the level of progesterone in Meishan sows was significantly higher than that in Yorkshire sows (*p* < 0.05). After day 18 of gestation, the level of progesterone in the two breeds tended to be identical. In Meishan pigs, progesterone levels on day 32 of pregnancy were significantly lower than that on day 18 of pregnancy and had an opposite difference with *XLOC-2222497* and *AKR1C1*. Meanwhile, the porcine endometrial epithelial cells were treated with different concentrations of progesterone (P4) for 48 h. As shown in Figure 6B, 100 nmol/L P4 treatment significantly promoted the expression levels of *XLOC-2222497*. P4 treatment in porcine endometrial epithelial cells also promoted the expression of *AKR1C1* (Figure 6C). The Western blot results were consistent with qRT-PCR results (Figure 6D). The above results demonstrated the regulation of progesterone on *XLOC-2222497* and *AKR1C1*.

## 3. Discussion

Progesterone, a natural female hormone, is an essential hormone for pregnancy [46,47]. AKR1C1 belongs to the aldo-keto reductase (AKR) superfamily of nicotinamide adenine dinucleotide phosphate (NADPH)-dependent oxidoreductases [48,49] and has a major role in progesterone metabolism [40,47,50]. Moreover, AKR1C1 can bind to the promoter region of the progesterone receptor and hereby decreases receptor activity [51]. AKR1C1 is expressed ubiquitously [52,53]. Our qRT-PCR result also confirmed the ubiquitous expression of AKR1C1. In steroidogenic tissues, AKR1C1 catalyzes the final steps in progesterone biosynthesis. In peripheral tissues, including steroid hormone target tissues, AKR1C1 converts progesterone to its inactive form of 20-alpha-hydroxy-progesterone and regulates the amount of hormone that can bind to members of the nuclear receptor superfamily, ultimately regulating gene expression [50]. Recent studies showed that AKR1C1 could induce signal transducer and activator of transcription (STAT) activation [54,55], which was involved in pregnancy [56]. In this study, the expression level of progesterone in the serum of pregnant sows decreased from days 18 to 32 of pregnancy, and AKR1C1 was highly expressed on day 32 of pregnancy. These results were consistent with previous studies that AKR1C1 played a critical role in controlling the progesterone concentration [47,57,58,59].

To explore the transcriptional regulation of AKR1C1, luciferase activity assay was performed and demonstrated that the AKR1C1 core promoter region was located in the region from −706 to −564. In this region, there was one CTCF binding site and two AREs. The site-mutation experiment showed that ARE was important for the promoter activity of AKR1C1. NRF2 is a redox-regulated transcription factor that coordinates the basal and inducible expression of a vast array of cytoprotective and antioxidant genes through binding to ARE [60,61,62]. Previous studies also confirmed that NRF2 was a regulator of the AKR1C family via direct binding to the ARE located in the promoter regions of the AKR1Cs [49,63,64]. Wentilactone A (WA) inhibited the expression of AKR1C1 via the insulin like growth factor 1 receptor (IGF-1R)/ insulin receptor substrate 1 (IRS-1)/ phosphatidylinositol 3-kinase (PI3K)/ protein kinase B (AKT)/NRF2 signaling pathway [43]. Accumulation of p62 inhibits Keap1-mediated NRF2 protein degradation by competing with NRF2 for the binding site on Kelch-like enyol-CoA hydratase (ECH)-associated protein 1 (Keap1), a cytosolic repressor protein of NRF2, resulting in transcriptional upregulation of NRF2 downstream genes [65,66]. NRF2 also contains a phosphodegron, phosphorylation of which promotes nuclear export and a return to basal antioxidant signaling. A central regulator of NRF2 in this manner is glycogen synthase kinase 3β (GSK3β) [67]. GSK3β phosphorylates Fyn at the threonine residue. Phosphorylated Fyn accumulates in the nucleus and phosphorylates NRF2, and brings about the nuclear export of NRF2, resulting in NRF2 rebinding Keap1 and being rapidly degraded [68,69]. Wang et al. found the expression levels of AKR1C1 and NRF2 were elevated in progestin-resistant endometrial epithelia. The NRF2/AKR1C1 pathway may represent a new therapeutic strategy for treatment of endometrial hyperplasia/cancer [70].

Due to the powerful and diverse functions of long non-coding RNAs, a large number of lncRNAs have been found and identified. However, few studies focus on lncRNAs related to pig pregnancy. In porcine endometrial tissue, Wang et al. found several differentially expressed lncRNAs that may play a vital role in the process of implantation using RNA sequencing [71,72,73]. In this study, a novel lncRNA *XLOC-2222497* was screened by RNA-seq. The online software CPC was used to predict the coding potential of *XLOC-2222497* and showed no coding potential. Cell-fractionation assay and RNA FISH results demonstrated that *XLOC-2222497* was mainly located in the nucleus. Then the *XLOC-2222497* overexpression and knockdown results showed the positive regulation of *XLOC-2222497* on *AKR1C1*. Furthermore, *XLOC-2222497* and *AKR1C1* were both highly expressed on day 32 of pregnancy, which was consistent with their positive regulation. lncRNAs can regulate the expression of target genes via a variety of mechanisms [74]. Because XLOC-2222497 was mainly located in the nucleus, there were two possible molecular mechanisms underlying the regulation of *XLOC-2222497* on *AKR1C1*: (1) *XLOC-2222497* increased the stability of *AKR1C1* mRNA. (2) *XLOC-2222497* recruited transcription factor to the promoter region of the *AKR1C1* gene to promote the transcription of *AKR1C1*. The specific molecular mechanism needs further studies. In addition, one of the most notable features of lncRNAs is their tissue specificity as compared to protein coding genes [75,76]. The tissue expression profile revealed that XLOC-2222497 was highly expressed in the endometrium, which was consistent with the tissue specificity of lncRNAs. To our knowledge, *XLOC-2222497* is the first identified lncRNA regulating *AKR1C1.*

**Figure 7:**
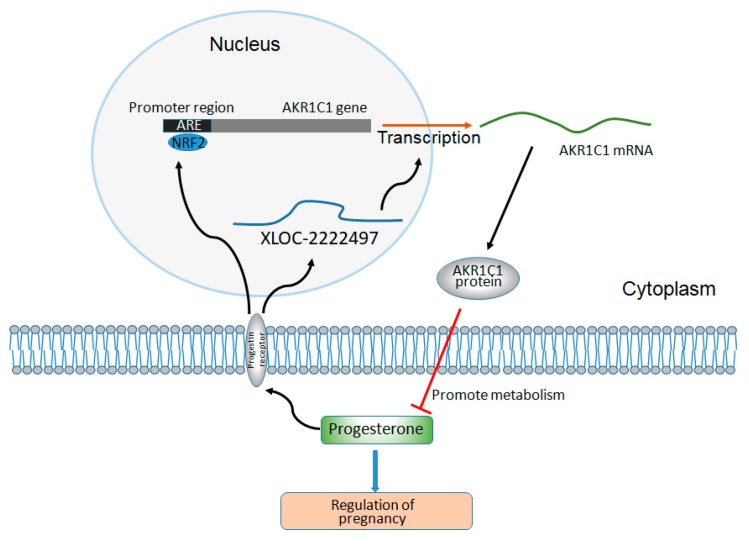
The regulation diagram of *XLOC-2222497, AKR1C1*, and progesterone.

Moreover, the expression level of *AKR1C1* and *XLOC-2222497* in endometrial cells increased significantly after the treatment of progesterone. This result was consistent with the previous result [77]. The administration of progesterone might activate the NRF2/ARE signal pathway [78]. Ghadiri et al. found that progesterone at both 16 and 32 mg/kg doses induced expression of NRF2 [79]. Byrne et al. revealed norgestrel, an FDA-approved synthetic analog of progesterone, inhibited GSK3β and modulated NRF2 expression at the post-translational level, bringing about its phosphorylation, and subsequent translocation into the nucleus where it bound antioxidant response elements (AREs), bringing about the upregulation of various antioxidants, and detoxifying and cytoprotective genes [69,80,81]. Moreover, cytoplasmic NRF2 expression is significantly correlated with the expression of progesterone receptor (PR), which suggested a possible functional interaction between NRF2 and PR [51]. Taken together, it might corroborate a possible feedback regulation mechanism which merits further investigations: the high progesterone level leads to the increase of *XLOC-2222497* and NRF2 by PR, and thus activates AKR1C1, metabolizing progesterone to its inactive form and maintaining a mutual balance (Figure 7). P4-mediated *XLOC-2222497* and *AKR1C1* expression in the endometrium was conducive to maintaining the stability of the intrauterine environment. In addition, these effects should be also taken into account when exogenous progesterone is administered. The above results demonstrated the important roles of the *XLOC-2222497*/*AKR1C1*/progesterone signaling pathway in pregnancy and provided new potential targets for pregnancy maintenance and its control.

## 4. Materials and Methods

### 4.1. Ethics Statement

In our research, all animal procedures were approved by The Scientific Ethics Committee of Huazhong Agricultural University, Wuhan, China (ID number: HZAUSW2015-017, permitted at February 2015).

### 4.2. Animal Sources and Sample Collection

The experimental populations consisted of four Yorkshire pigs and four Meishan pigs of similar age with genetic background from one commercial herd. All pigs were raised under the same conditions and under a standardized feeding regimen with free access to water. Each breed was randomly assigned to two groups: day 18 (*n* = 2) and day 32 (*n* = 2), and was artificially inseminated at the same time. Uteri were obtained from animals slaughtered on days 18 and 32 of pregnancy. Each uterine horn was flushed with sterile phosphate buffer saline (PBS) (pH 7.4), and subsequently opened longitudinally on the inner side. Samples from the endometrium of Meishan pigs and Yorkshire pigs on days 18 and 32 of pregnancy were taken. Tissue samples were frozen in liquid nitrogen and stored at −80 °C before RNA isolation. All animal procedures were approved by The Scientific Ethics Committee of Huazhong Agricultural University, Wuhan, China (ID number: HZAUSW2015-017, permitted at February 2015).

### 4.3. Library Construction and Sequencing

Total RNA was extracted from the endometrium using the TRIzol reagent (Invitrogen, Life Technologies, CA, USA). The RNA samples were quantitated and subjected to quality inspection. Total DNA was extracted using the TIANamp Genomic DNA kit (Tiangen, CA, China) according to the manufacturer’s protocol.

### 4.4. Identification of Differentially Expressed Genes (DEGs)

First, the expression level of each gene was calculated using the HTseq software (0.6.1) [82], and was normalized based on the reads per RPKM method [83]. Subsequently, DEGs were identified using the R packages DEGseq (1.18.0) [84]. The RNA-seq data was deposited in the Gene Expression Omnibus (accession GSE141564).

### 4.5. Quantitative Real-Time PCR for Gene Expression

MiRNAs and mRNAs were reverse transcribed using the RevertAid First Strand cDNA Synthesis Kit (Thermo, Wuhan, China) in accordance with the manufacturer’s instructions. qPCR was performed using a standard UltraSYBR Mixture (CWBIO, Beijing, China) in the Roche LightCyler 480 system (Roche, Mannheinm, Germany) according to the manufacturer’s instructions. Porcine *β-actin* gene was used as the endogenous control gene for mRNA RT-PCR. The RT-PCR data were analyzed using the 2^-∆∆CT^ method, as previously described [85]. The relative fold changes of mRNA expression were quantified by normalizing the cycle threshold (CT) value of the experimental gene to the mean CT value of the control β-actin gene.

### 4.6. Western Blot

Cells were split by radioimmunoprecipitation assay (RIPA) buffer (Beyotime, Jiangsu, China) and supplemented with 0.01% of phenylmethanesulfonyl fluoride (PMSF) (Beyotime, Jiangsu, China). The protein was separated by sodium dodecyl sulfate (SDS) polyacrylamide gel electrophoresis, and then was transferred to a polyvinylidene fluoride (PVDF) membrane (Millipore, Boston, USA). Next, the protein was incubated with the corresponding primary antibody and secondary antibody. Antibodies included AKR1C1 (1:1000, ABclonal, Wuhan, China), and β-actin (1:1000, Boster, Wuhan, China).

### 4.7. Cell Culture and Transfection

Primary endometrial cells were isolated from the uteri of Yorkshire pigs. For in vitro culture of endometrial cells, PBS (pH = 7.4) was used to wash the external surface of the uteri. When opened longitudinally, the endometrium was separated from the myometrium by using a sterilized blade. The endometrium tissues were collected and flushed with PBS (pH = 7.4). Minced tissues mixed with Dulbecco’s modified eagle medium/Nutrient mixture F-12 (DMEM/F-12) (Hyclone, Logan, USA) culture medium containing 0.1% collagenase I were placed in a 5% CO_2_ incubator at 37 °C for 2.5 h, and the mixture was shaken every half hour. The DMEM/F-12 culture medium containing 10% fetal bovine serum (FBS) (CLARK, Richmond, USA) supplemented with 1% penicillin–streptomycin was used to terminate the digestion. Afterwards, the mixture was filtered by using the sterilized cell screen and the solution was transferred to the tube. The precipitates were collected to release the endometrial cells after twice centrifugation. The porcine endometrium cells were maintained at 37 °C in a humidified atmosphere containing 5% CO_2_ in Dulbecco’s modified Eagle medium (Hyclone, Logan, USA) supplemented with 10% fetal bovine serum (FBS) (CLARK, Richmond, USA). Epithelial and stromal cells were isolated by the differential adhesion method. The purified epithelial cells were plated and grown until they reached 70–80% confluent. Then, overexpression plasmid and small interfering RNAs (siRNAs) were transfected using lipofectamine 2000 (Invitrogen, Carlsbad, CA, USA).

### 4.8. Immunohistochemistry

Partial samples of the endometrium from Meishan pigs and Yorkshire pigs were fixed in 4% formaldehyde solution to make a paraffin section. The immunohistochemistry analysis was performed following the procedures: xylene dewaxing, xylene replaced by gradient alcohol, treatment of peroxide, antigen repair, serum blocking, anti-AKR1C1 incubation, secondary antibody incubation, diaminobenzidine (DAB) coloration, hematoxylin staining, 0.5% hydrochloric acid differentiation, wash with water, gradient alcohol dehydration, xylene transparentization, and mounting. Stained slices were observed under a 400-fold microscope.

### 4.9. Endocrinologic Measurement

Progesterone levels of serum from Yorkshire pigs and Meishan pigs on days 9, 12, 15, 18, and 32 of pregnancy were measured by an enzyme linked immunosorbent assay (ELISA) kit (Fusheng, CA, China) according to the manufacturer’s protocol. The brief steps are described as follows: standard preparation, sample dilution and incubation, incubation of conjugate reagent, coloration, and spectrophotometry. There were three Yorkshire pigs and two Meishan pigs in each stage.

## 5. Conclusions

This study investigated long non-coding RNA regulation in the endometrium of Yorkshire and Meishan pigs at different days of pregnancy using high-throughput sequencing. We first identified an AKR1C1-related lncRNA in porcine endometrium. Further experimental results showed that *XLOC-2222497* regulated *AKR1C1* in porcine endometrial epithelial cells, thus participating in the regulation of progesterone metabolism. This study provides valuable information for future transcriptome studies of porcine endometrium, and the molecular regulation of pregnancy.

## Figures and Tables

**Table 1:** The predicted coding potential of *XLOC-2222497.*

Gene	Coding/Non-Coding	Coding Score
*XLOC-2222497*	Non-coding	-0.589266
*AKR1C1*	Coding	6.60874
*NEAT1*	Non-coding	-1.21743

(Coding score <0 means no coding potential; coding score >0 means certain coding potential).

## Supplementary Materials

The following are available online at https://www.mdpi.com/1422-0067/21/9/3232/s1. Supplementary Table S1: List of highly expressed lncRNAs with RPKM >100. Supplementary Table S2: Differentially expressed mRNA between YK32 and YK18. Supplementary Table S3: Differentially expressed lncRNA between YK32 and YK18.
