# Supplementary material for: The Interaction of lncRNA XLOC-2222497, AKR1C1, and Progesterone in Porcine Endometrium and Pregnancy
Source: Int J Mol Sci. 2020 May 2;21(9):3232. doi: 10.3390/ijms21093232 (PMC7247569; doi:10.3390/ijms21093232)
Supplement: Supplementary file 1 [file ijms-21-03232-s001.pdf]

Table S1. List of highly expressed lncRNAs with RPKM>100

DB1801	RPKM	DB1802	RPKM	DB3201	RPKM	DB3202	RPKM
XLOC_3841932	1514.36	XLOC_3841932	7212.09	XLOC_1890946	3757.31	XLOC_3841932	4309.99
XLOC_1938806	685.50	XLOC_2995004	1968.04	XLOC_3841932	2445.85	XLOC_1890946	2802.77
XLOC_2995004	684.33	XLOC_3841932	1314.75	XLOC_3841932	792.04	XLOC_3841932	762.10
XLOC_2219602	668.84	XLOC_2995004	1039.62	XLOC_2995004	653.51	XLOC_2995004	625.16
XLOC_543878	588.52	XLOC_2219602	994.45	XLOC_2995004	427.28	XLOC_2995004	435.70
XLOC_2221176	569.17	XLOC_1938806	660.05	XLOC_1938806	334.22	XLOC_2219602	394.52
XLOC_3841932	496.54	XLOC_2221176	567.32	XLOC_1822528	319.28	XLOC_1938806	349.68
XLOC_2995004	269.99	XLOC_543878	363.15	XLOC_2412402	203.52	XLOC_2221176	304.57
XLOC_2412402	169.56	XLOC_2412402	166.05	XLOC_543878	193.17	XLOC_1822528	230.80
XLOC_546566	124.84	XLOC_2831661	135.52	XLOC_2639652	181.45	XLOC_2412402	192.81
XLOC_3293032	118.43	XLOC_3293032	125.61	XLOC_2221176	143.93	XLOC_543878	180.81
XLOC_2831661	115.65	XLOC_2017489	122.83	XLOC_2219602	121.98	XLOC_2531070	109.46
XLOC_508308	104.66	XLOC_546566	122.40			XLOC_546566	104.80
XLOC_3038776	102.11	XLOC_2238416	104.08				
XLOC_1001671	101.39	XLOC_1001671	100.09				

MS1801	RPKM	MS1802	RPKM	MS3201	RPKM	MS3202	RPKM
XLOC_2219602	2181.00	XLOC_2219602	868.72	XLOC_3841932	4310.78	XLOC_3841932	1613.54
XLOC_3841932	1753.76	XLOC_3841932	686.86	XLOC_1890946	3380.87	XLOC_1890946	1227.03
XLOC_2995004	1625.38	XLOC_1938806	542.56	XLOC_3841932	1028.54	XLOC_2219602	1117.87
XLOC_2995004	1207.82	XLOC_543878	367.68	XLOC_2219602	775.10	XLOC_3841932	461.77
XLOC_543878	873.90	XLOC_3841932	342.23	XLOC_1822528	421.49	XLOC_1938806	346.97
XLOC_1938806	824.83	XLOC_2995004	278.67	XLOC_1938806	329.04	XLOC_2995004	304.04
XLOC_2221176	365.99	XLOC_2221176	189.70	XLOC_2995004	212.78	XLOC_2995004	261.23
XLOC_3841932	343.46	XLOC_2412402	181.16	XLOC_2995004	210.88	XLOC_2221176	165.55
XLOC_1491321	134.50	XLOC_2995004	179.01	XLOC_2412402	169.77	XLOC_2412402	141.48
XLOC_141729	112.37	XLOC_1890946	177.48	XLOC_2531070	160.47	XLOC_2531070	139.18
XLOC_911471	109.62	XLOC_546566	116.80	XLOC_2221176	148.67	XLOC_1822528	126.68
XLOC_2412402	108.27	XLOC_665343	101.66	XLOC_2017489	117.39	XLOC_543878	100.06
XLOC_018738	105.54						
XLOC_2540878	102.26						
